# Post-acute exercise cardiovagal modulation in older male adults with and without type 2 diabetes

**DOI:** 10.1007/s00421-023-05357-3

**Published:** 2023-12-20

**Authors:** João Luís Marôco, Inês Arrais, Tiago Silvestre, Marco Pinto, Sérgio Laranjo, João Magalhães, Helena Santa-Clara, Bo Fernhall, Xavier Melo

**Affiliations:** 1 Integrative Human Physiology Laboratory, Manning College of Nursing & Health Sciences, University of Massachusetts Boston, Boston, MA USA; 2 Ginásio Clube Português, Research & Development Department, GCP Lab, Lisbon, Portugal; 3 Centro Interdisciplinar de Estudo da Performance Humana, Faculdade de Motricidade Humana—Universidade de Lisboa, Oeiras, Portugal; 4 Faculdade de Ciências da Saúde e do Desporto, Universidade Europeia, Lisbon, Portugal; 5 Department of Physiology, NOVA Medical School, Faculdade de Ciências Médicas, NMS, FCM, Universidade NOVA de Lisboa, Lisbon, Portugal; 6 Comprehensive Health Research Center. NOVA Medical School, Faculdade de Ciências Médicas, NMS, FCM, Universidade NOVA de Lisboa, Lisbon, Portugal; 7 Centro de Investigação Interdisciplinar Egas Moniz (CiiEM), Egas Moniz School of Health and Science, Caparica, 2829-511 Almada, Portugal

**Keywords:** Type 2 diabetes, Aging, Cardiac autonomic dysfunction, Baroreflex, Acute exercise

## Abstract

**Purpose:**

We examined heart rate variability (HRV) and baroreflex sensitivity (BRS) disease- and age-related response at 10-and 60-min after an acute high-intensity interval (HIIE) and moderate continuous exercise (MICE) in older adults with and without type 2 diabetes mellitus (T2DM) and healthy young adults.

**Methods:**

Twelve older male adults with (57–84 years) and without T2DM (57–76 years) and 12 healthy young male adults (20–40 years) completed an isocaloric acute bout of HIIE, MICE, and a non-exercise condition in a randomized order. Time and Wavelets-derived frequency domain indices of HRV and BRS were obtained in a supine position and offline over 2-min time-bins using Matlab.

**Results:**

HIIE but not MICE reduced natural logarithm root mean square of successive differences (Ln-RMSSD) (*d* = − 0.85; 95% CI − 1.15 to − 0.55 ms, *p* < 0.001), Ln-high-frequency power (*d* = − 1.60; 95% CI − 2.24 to − 0.97 ms^2^; *p* < 0.001), and BRS (*d* = − 6.32; 95% CI − 9.35 to − 3.29 ms/mmHg, *p* < 0.001) in adults without T2DM (averaged over young and older adults without T2DM), returning to baseline 60 min into recovery. These indices remained unchanged in older adults with T2DM after HIIE and MICE. Older adults with T2DM had lower resting Ln-RMSSD and BRS than aged-matched controls (Ln-RMSSD, *d* = − 0.71, 95% CI − 1.16 to − 0.262 ms, *p* = 0.001; BRS d = − 3.83 ms/mmHg), 95% CI − 6.90 to − 0.76,* p* = 0.01).

**Conclusions:**

Cardiovagal modulation following acute aerobic exercise is intensity-dependent only in adults without T2DM, and appears age-independent. These findings provide evidence of cardiac autonomic impairments in older adults with T2DM at rest and following aerobic exercise.

**Supplementary Information:**

The online version contains supplementary material available at 10.1007/s00421-023-05357-3.

## Introduction

Cardiac autonomic dysfunction is characterized by diminished cardiovagal predominance at rest coupled with parallel increases in cardiac sympathetic modulation (Tarvainen et al. 2014). Importantly, this cardiac autonomic imbalance at rest likely precedes the onset of cardiac autonomic neuropathy (CAN), which is a commonly overlooked complication in older adults with T2DM associated with a higher risk of sudden cardiac death and all-cause mortality (Rathmann et al. 1993; Maser et al. 2003; Huggett et al. 2005). Low cardiovagal baroreflex sensitivity (BRS) may account for cardiac autonomic dysfunction in older adults with T2DM, as it limits the ability to reflexively increase vagal and inhibit sympathetic outflows via the nucleus tractus solitarius, in response to transient increases in arterial pressure (Weston et al. 1996; Heusser et al. 2005).

Consistent with the reactive hypothesis, the acute exercise model provides clinical insights into cardiac autonomic stress reactivity as it uncovers abnormalities that are not present at rest following both submaximal and maximal exercise (Figueroa et al. 2006; Banthia et al. 2013; Verma et al. 2018; Bhati et al. 2019; Goldberger et al. 2022). As such, exercise testing-based methods using indices of heart-rate variability (HRV) were recently proposed to be more precise and relevant in diagnosing CAN in T2DM compared to resting assessments (Bhati et al. 2019). T2DM may alter the cardiovagal response to acute exercise, such that decreases immediately after exercise testing (Figueroa et al. 2006; Banthia et al. 2013; Verma et al. 2018; Bhati et al. 2019; Goldberger et al. 2022), or the more pronounced reductions with high rather than moderate-intensity exercise in healthy young and older adults, may not be observed (Kaikkonen et al. 2008; Niemela et al. 2008; Stanley et al. 2013; Michael et al. 2016, 2017a, b; Venturini et al. 2016). Nonetheless, and to the best of our knowledge, this has never been previously investigated.

Therefore, this study aimed to examine HRV and BRS disease- and age-related response patterns at 10 and 60 min after an acute bout of high-intensity interval exercise (HIIE) and moderate-intensity continuous exercise (MICE) in older adults with and without T2DM and healthy young adults. We hypothesized that (1) reductions in post-exercise HRV and BRS would not be observed in older adults with T2DM compared to those without, and that (2) declines in HRV and BRS estimates following HIIE would be more pronounced compared to MICE.

## Methods

### Participants

A detailed schematic of participant recruitment and enrolment for the study is provided in Supplement 1. The T2DM group included 12 male older adults (aged 57–84 years) with long-standing [(17 (6) years], insulin and metformin-treated (67%) T2DM as diagnosed by the criteria of the American Diabetes Association [fasting HbA1 = 7.25% (0.73)] (Care and Suppl 2022). Exclusion criteria were as follows: smoking, cardiac (e.g., heart failure, ischemic heart disease), renal or musculoskeletal diseases. In addition, 24 healthy male participants were assigned to the comparison groups of young (*n* = 12; aged 20 to 40 years) and older adults (*n* = 12; aged 57 to 76 years) without T2DM. All participants completed the Physical Activity Readiness Questionnaire for Everyone (PAR-Q +) and the International Physical Activity Questionnaire (IPAQ). Participants without T2DM were active as they accumulated at least 150 min wk^−1^ of moderate to vigorous physical activity (M = 200, SD = 25 min wk^−1^), while participants with T2DM were inactive (M = 65, SD = 30 min wk^−1^). Exclusion criteria for participants without T2DM were as follows: smoking, having cardiac (e.g., heart failure, ischemic heart disease), renal or musculoskeletal diseases, or taking cardioactive medication. All participants gave written informed consent after a detailed explanation of the experimental procedures and aims of the study. All experimental procedures were approved by the ethics committee of Faculdade de Motricidade Humana – Universidade de Lisboa (10/2020) and were aligned with both the Declaration of Helsinki for Human Research and the Ethical Standards in Sport and Exercise Science Research: 2020 Update (Harriss et al. 2019).

### Experimental design

The study was designed as a randomized, cross-over, repeated-measures experiment. Participants attended 3 separate intervention sessions consisting of an acute bout of HIIE, an acute bout of MICE, or no exercise (CON) in a randomized order (http://www.randomizer.org/) to which participants were blinded until arrival at the laboratory. On a separate day before interventions, all participants performed a cardiopulmonary exercise test (CPET) and body composition was also measured with a medical bioimpedance device (seca mBCA 515, Hamburg, Germany). Exercise sessions began with a 3 min warm-up at 60% heart reserve (HRR) and ended with an identical cool-down. Both the HIIE and MICE protocols were designed to match a weekly energy expenditure (EE) of 8 kcal.kg^−1^ week^−1^ (Ramírez-Vélez et al. 2019) comprising 3 hypothetical exercise sessions per week. Both exercise protocols were individually tailored to each participant regarding their weight and peak oxygen uptake (V̇O_2 peak_), and the prescribed target intensities were supervised by an exercise physiologist with a heart rate monitor (Garmin, US) throughout the session. To account for possible diurnal variations, each participant performed all interventions at the same time of the day (in the mornings), with a minimum of 48 h between sessions. Post-exercise measurements at 10 and 60 min were aimed to characterize the post-exercise biphasic response of the autonomic nervous system (Michael et al. 2017a). Participants reported to the laboratory in a fasted state (≥ 4 h) and refrained from vigorous exercise, vitamin supplements, foods/beverages containing caffeine, and alcohol for at least 12 h before each session.

### Interventions

The HIIE comprised 1 min exercise bouts at 90% of V̇O_2 Reserve_ interspersed by 1 min active recovery bouts at 60% V̇O_2 Reserve_ (1:1). Resting oxygen uptake (V̇O_2 Rest_) was assumed to be one metabolic equivalent (3.5 mL kg min^−1^) for all participants. The number of bouts of exercise recovery for each participant was tailored to achieve the desired EE. An example is a participant with a V̇O_2 peak_ of 30 mL kg^−1^ min^−1^, weighing 80 kg, and considering that 5 kcal are expended for every 1L of O_2_ consumed, would need 6 bouts to match the targeted EE. This protocol was performed on a motorized treadmill and the participants walked or ran at the pace required to achieve the target intensity.

The MICE protocol intensity was set at 60% of V̇O_2 Reserve_ and the duration was adjusted so that each participant achieved the targeted EE. An example is a participant with a V̇O_2 peak_ of 30 mL kg^−1^ min^−1^, weighting 80 kg, and considering that 5 kcal are expended for every 1L of O_2_ consumed, would exercise for 21 min to match the required EE.

### Evaluation of cardiac autonomic function

#### Heart rate variability

Participants were assessed in a rested supine position in a quiet climate-controlled room (22–24 ℃) during all repeated measurements. The R–R intervals were sampled at 300 Hz frequency to acquire a digital sequence of R waves using the 5-ECG lead module of the Finapres Nova device (Finapres® NOVA, Finapres Medical Systems, Amsterdam, The Netherlands). All data acquisition and offline analyses were performed following the standards of the Task Force of the European Society of Cardiology and the North American Society of Pacing and Electrophysiology (Task Force of the European Society of Cardiology and the North American Society of Pacing and Electrophysiology 1996).

#### Heart rate variability data analysis

All HRV analyses were performed offline using the *FisioSinal* software built-in Matlab (Tavares et al. 2011). Following R-R peak detection and semiautomatic removal of signal artifacts, 2 min time series were constructed using a cubic spline interpolation which allowed estimation of both time-domain and spectral power indices (Tavares et al. 2011; Wu et al. 2020). Ectopic heartbeats (M = 1, SD = 6 b.min^−1^) were also excluded from the final analysis. The time-domain indices used to characterize HRV were the standard deviation of NN intervals (SDNN)—a measure of overall variability, and the root mean square of the sum of the squares of the differences between NN intervals (RMSSD)—a measure of cardiovagal modulation, both in milliseconds. Additionally, non-linear time-domain parameters were derived from the Poincaré plot including the vertical deviation (SD1), the longitudinal deviation (SD2), and the non-linear ratio SD1/SD2. Analyzing HRV through non-linear methods is important since R–R intervals are often unpredictable due to the complex interactions of the diverse mechanisms that regulate HRV (e.g., central and autonomic neural regulations, humoral, hemodynamic) (Shaffer and Ginsberg 2017). The time–frequency domain analysis was conducted using the Daubechy-12 discrete wavelet algorithm, which allowed the estimation of low (0.04 to 0.15 Hz) and high-frequency bands (0.15 to 0.4 Hz) in absolute and normalized power units. The low-frequency bands reflect both vagal and sympathetic modulation to the heart, whereas the high frequencies reflect only cardiovagal modulation (Shaffer and Ginsberg 2017). Wavelet analysis was chosen instead of fast Fourier transform as it is better suited to characterize acute responses of the autonomic nervous system during the post-exercise period (Belova et al. 2007).

#### Cardiovagal baroreflex sensitivity

The spontaneous sequence method, chosen for its non-invasive nature, was employed to estimate the baroreflex effectiveness index (BEI) and the gain of the baroreflex arc (i.e., baroreflex sensitivity)(La Rovere et al. 2008). Briefly, we utilized the automated baroreflex module of *FisioSinal* (Tavares et al. 2011), which identifies systolic blood pressure and RR interval ramps defined as adjacent oscillations of at least > 1 mmHg and > 4 ms, respectively. Beat-to-beat SBP was recorded using finger plethysmography (Finapres® NOVA, Finapres Medical Systems, Amsterdam, The Netherlands). A BRS event was defined as the overlap between the BP ramps and the concordant changes in RR. Thus, BRS was defined as the average of BRS slopes. The baroreflex effectiveness index was calculated as the total number of BRS events divided by the total number of BP ramps observed during the 2 min time-bin.

#### Reliability of cardiovagal modulation and baroreflex sensitivity

Our laboratory has excellent intra- and inter-day reliability for linear time-domain (Ln-RMSSD; ICC: 0.98; 95% CI 0.97–0.99 and ICC: 0.95; 95% CI 0.90–0.97, respectively) and spectral power analyses (Ln-HF; ICC: 0.97; 95% CI: 0.96 to 0.99 and ICC: 0.93; 95% CI 0.87–0.96, respectively). Intra- and inter-day reliability for BRS is considered good (ICC: 0.87; 95% CI 0.77–0.93 and ICC: 0.78; 95% CI 0.61–0.89, respectively).

### Cardiopulmonary exercise testing

An incremental cardiopulmonary exercise test with mixing-chamber gas exchange measurements (K5, Cosmed, Rome, Italy) was conducted on a motorized treadmill. Participants were tested 4 h post-prandial and under regular medication. Blood pressure was assessed by auscultation using an aneroid sphygmomanometer, and heart electrical activity was continuously monitored through a 12-lead electrocardiogram (at rest, at the end of each stage, and every min after peak effort). A certified physician supervised the protocol, which started with an initial walking period (3 min) at a comfortable self-selected pace (a pace that could be sustained for 20–25 min). Subsequently, the grade was set to 5% and increased by 1% every 2 min until volitional exhaustion (Porszasz et al. 2003). The recovery phase consisted of a 3 min walking period. The increases in velocity were simultaneous with those of grade and were matched for 25 W increments according to the following equation: $WR=m\times g\times v\times \mathrm{sin}\left(\mathrm{\alpha }\right)$, where WR (watts) is work rate; m is body mass (kg); g is the gravitational acceleration (9.81 ms^−1^), is the velocity (m s^−1^), and *α* is the grade. The O_2_ and CO_2_ analysers were calibrated using ambient air and normal calibration gases with known concentrations before each examination (16.7% O_2_ and 5.7% CO_2_). The turbine flowmeter of the K5 Cosmed was calibrated using a 3-L syringe according to the manufacturer’s guidance. Participants were encouraged to exercise until exhaustion, as defined by ≥ 2 of the following criteria: attaining a plateau (variance of < 2.1 mL kg^−1^ min^−1^ during the last 60-s of the test); HR_peak_ ≥ 90% of the age-predicted (208–0.7 × age); respiratory exchange ratio (RER) > 1.10; rating perceived exertion (RPE) ≥ 18 (Borg 6–20); subjective judgment by the observer that the participant can no longer continue, even after encouragement, unless clinical criteria for early; test termination were observed. Pulmonary gases were time averaged over 15-s time bins and the V̇O_2 peak_ was defined as the highest V̇O_2_ value during the final 20-s of exercise. Heart rate recovery (HRR1) was also calculated as the difference between HR_peak_ and HR at the first minute following maximal exercise testing.

### Anthropometrics, body composition

Body composition parameters (fat mass and fat-free mass) were estimated using a bioimpedance device (seca mBCA 515, seca gmbh & co. kg, Hamburg, Germany) featuring four pairs of electrodes positioned at each hand and foot that allow impedance to be measured with a current of 100 μA at frequencies between 1 and 1 000 kHz. Height and body weight were measured to the nearest 0.1 cm and 0.1 kg on a scale with an attached stadiometer (model 770, Seca, Hamburg, Deutschland).

### Statistics analysis

Based on a standardized medium effect size of 0.25, the a priori power analysis (G-Power Version 3.1.9.3) suggested that a total of 36 participants were required to detect significant differences between interventions and groups (*α* = 0.05, 1 − *β* = 0.8).

All statistical analyses were conducted using R software, version 4.0.1 (R Core Team 2020), with a significant level (*α*) of < 0.05. Data are presented as mean (SD) unless otherwise noted. The normality and homoscedasticity assumptions were verified with the Shapiro–Wilk and Levene tests, respectively, and by visual plot inspection. A natural logarithm (Ln) transformation was applied to the HRV indices, as these were not normally distributed. One-way ANOVA with Tukey’s HSD correction were used to compare the characteristics of the participants and aerobic exercise intensities.

Intra- and inter-day relative reliability was assessed with a two-way absolute agreement mixed model intraclass correlation coefficient (ICC (2,1)) computed with irr package, using three static repeated measurements. The ICC was interpreted as: poor [< 0.50], moderate [0.50, 0.75], good [0.75, 0.90], and excellent [> 0.90] (Koo and Li 2016).

The changes in main cardiovagal indices (e.g., Ln-RMSSD, Ln-HF, BRS) from pre- to post- conditions, were examined using linear mixed models fitted with restricted maximum likelihood and applying Satterthwaite's method for approximating degrees of freedom for the F test from the lmerTest package (Kuznetsova et al. 2017). Fixed effects were defined as time, intervention, and group, and the random intercept was defined as each participant. Partial omega squares (*ω*^*2*^) were calculated for main effects and interactions (condition*time; group*time; group*condition and condition*time*group) using sjstats package and interpreted applying the rough benchmarks set by Cohen (1988) [small (*ω*^*2*^ < 0.05), medium (*ω*^*2*^ < 0.25), and large *ω*^*2*^ > 0.25) effects sizes]. V̇O_2 peak,_ HR_peak,_ HR, and %fat mass were added one-by-one to the mixed model as covariates. Post hoc comparisons were performed using Tukey’s HSD test with the emmeans package, in the presence of significant differences in the main effects and interactions. As similar cardiovagal response patterns after acute aerobic exercise were observed in young and older adults, despite differences at rest, post hoc comparisons were averaged over these two levels and compared to those of participants with T2DM.

## Results

### Characteristics of the participants

Clinical and demographic characteristics are depicted in Table 1. Participants with T2DM had a higher BMI [*F* (2, 33) = 6.97, *p* = 0.002, *ω*^2^ = 0.45] compared to young (*d* = − 5.0; 95% CI − 1.6 to − 8.4 kg m^−2^; *p* = 0.002), but not older adults (*d* = − 3.1; 95% CI − 6.6 to 0.3 kg m^−2^, *p* = 0.08). HR_peak_ [*F* (2, 33) = 45.40, *p* < 0.001, *ω*^2^ = 0.71] and V̇O_2 peak_ [*F* (2, 33) = 85.55, *p* < 0.001, *ω*^2^ = 0.82] were lower in participants with T2DM when compared to young (*d * = − 31.44, 95% CI − 28.70 to − 16.70 mL kg min^−1^, *p* < 0.001; *d* = − 46, 95% CI − 58 to − 34 b.min^−1^, *p* < 0.001) and older adults (*d* = − 8.78, 95% CI − 14.90 to − 2.70 mL.kg.min^−1^, *p* = 0.003; *d* = − 15, 95% CI − 27 to − 3 b min^−1^, *p* = 0.01). A lower HRR1 was observed in participants with T2DM [*F* (2, 33) = 4.08,* p* = 0.03, *ω*^2^ = 0.15] in comparison to young (*d* = − 10, 95% CI − 18 to − 1 b min^−1^, *p* = 0.02), but not older adults (*d* = − 7, 95% CI − 2 to 16 b min^−1^, *p* = 0.16).

**Table 1:** Characteristics of the participants

Characteristic	Young adults (*n* = 12)	Older adults (*n* = 12)	T2DM (*n* = 12)	*p* value^1^
Age (years)	27 (4)	64 (5)^#^	67 (8)^#^	< 0.001
Height (m)	1.75 (0.05)	1.74 (0.07)	1.68 (0.05)^#,^*	0.01
Weight (kg)	75.8 (7.9)	81.2 (10.2)	84.2 (15.0)	0.2
Body mass index (kg m^−2^)	24.8 (2.4)	26.7 (2.1)	29.8 (4.8)^#^	0.003
Waist circumference (m)	0.8 (0.1)	1.0 (0.1)^#^	1.0 (0.1)^#^	< 0.001
Fat mass (%)	17.5 (5.9)	25.0 (4.8)^#^	32.3 (5.5)^#,^*	< 0.001
Fat-free mass (kg)	62.2 (4.8)	60.8 (8.3)	56.5 (7.9)	0.14
bSBP (mmHg)	129 (10)	121 (16)	132 (12)	0.14
bDBP (mmHg)	78 (10)	74 (12)	77 (10)	0.6
HR_resting_ (b min^−1^)	60 (8)	60 (10)	65 (8)	0.086
HR_peak_ (b min^−1^)	188 (9)	157 (11)^#^	142 (116)^#,^*	< 0.001
HRR1 (b min^−1^)	24 (12)	21 (7)	14 (5)	0.026
V̇O_2 peak_ (L min^−1^)	4.2 (0.4)	2.7 (0.6)^#^	2.0 (0.5)^#,^*	< 0.001
V̇O_2 peak_ (mL kg^−1^ min^−1^)	55.5 (7.2)	32.8 (6.5)^#^	24.0 (4.2)^#,^*	< 0.001

Data presented as mean (SD)

*bSBP* brachial systolic blood pressure, *bDBP* brachial diastolic blood pressure, *HR* heart rate, *HRR1* heart rate recovery following the first minute after cardiopulmonary exercise testing, $\dot{V}{O}_{2} peak$ oxygen uptake

^1^One-way ANOVA with Tukey’s HSD correction for multiple testing

^#^Significant difference from Young Adults

*Significant difference from Older Adults

### Aerobic exercise characteristics

Isocaloric sessions of HIEE and MICE differed among groups for the total number of bouts ([*F* (2, 32) = 48.49, *p* < 0.001, *ω*^2^ = 0.73) and duration (*F* (2, 32) = 44.79, *p* < 0.001, *ω*^2^ = 0.71), but not total EE [*F* (2, 31) = 2.01, *p* = 0.15]. Participants with T2DM completed the highest number of HIIE bouts [12 (3)] and exercised for longer in MICE [34 (6) min], followed by the elderly [8 (2) bouts; 26 (5) min], and young adults [4 (1) bouts; 16 (2) min]. The peak HR during the last bout of HIIE [*F* (2, 32) = 36.65, *p* < 0.001, *ω*^2^ = 0.67] and mean HR during MICE [*F* (2, 32) = 27.80, *p* < 0.001, *ω*^2^ = 0.60] were higher in young [HIIE: 176 (10); MICE: 138 (7) b.min^−1^] compared to older adults [HIIE: 148 (12); MICE: 118 (7) b min^−1^], and lowest in participants with T2DM [HIIE: 132 (16); MICE: 113 (11) b min^−1^].

### Cardiovagal modulation and baroreflex sensitivity at rest

Indices of cardiovagal modulation differed among groups, as participants with T2DM had lower Ln-RMSSD [*F*(2, 33) = 34.61, *p* < 0.001, *ω*^2^ = 0.59], and Ln-HF [*F*(2, 33) = 25.37,* p* < 0.001, *ω*^2^ = 0.60] in comparison to young (Ln-RMSSD, *d* = − 1.25, 95% CI − 1.70 to − 0.80 ms, *p* < 0.001; Ln-HF, *d* = − 2.52, 95% CI − 3.39 to − 1.65 ms^2^,* p* < 0.001) and older adults (Ln-RMSSD, *d* = − 0.71, 95% CI − 1.16 to − 0.262 ms, *p* = 0.001; Ln-HF, *d* = − 1.36, 95% CI − 2.23 to − 0.50 ms^2^, *p* = 0.002; Tables 2 and 3). Similar results were observed for Ln-SDNN and Ln-SD1.

**Table 2:** Acute effects of aerobic exercise on heart rate variability—time domain

	Young adults			Older adults			T2DM			Time	Ex	Group	Ex*Time	Ex*time*Gr
	CON	HIIE	MICE	CON	HIIE	MICE	CON	HIIE	MICE	*p *(*ω*^2^)	*p *(*ω*^2^)	*p *(*ω*^2^)	*p *(*ω*^2^)	*p *(*ω*^2^)
Ln-IBI, ms										< 0.001	< 0.001	0.06	< 0.001	0.128
Pre	6.92 (0.15)	6.95 (0.13)	6.92 (0.11)	6.99 (0.13)	6.96 (0.13)	6.92 (0.15)	6.80 (0.13)	6.80 (0.14)	6.81 (0.17)	0.14	0.13	0.10	0.08	0.00
Post 10	6.89 (0.13)	6.48 (0.63)*^,‡,^^#^	6.78 (0.14)	7.00 (0.11)	6.69 (0.14)*^,‡,#^	6.80 (0.14)	6.82 (0.14)	6.63 (0.15)*^,‡^	6.52 (0.65)*^,‡^					
Post 60	6.92 (0.14)	6.79 (0.12)	6.90 (0.11)	6.97 (0.11)	6.76 (0.17)	6.84 (0.14)	6.82 (0.14)	6.68 (0.16)	6.75 (0.15)					
Ln-SDNN, ms										< 0.001	< 0.001	< 0.001	< 0.001	0.009
Pre	3.96 (0.33)	3.90 (0.60)	4.19 (0.46)	3.57 (0.31)	3.70 (0.39)	3.50 (0.52)	2.95 (0.51)	2.88 (0.50)	3.01 (0.40)	0.07	0.12	0.57	0.06	0.05
Post 10	4.21 (0.42)	3.47 (0.50)*^,‡,#^	3.90 (0.48)	3.72 (0.30)	3.05 (0.65)*^,‡,#^	3.36 (0.56)	2.91 (0.47)	2.64 (0.43)	2.96 (0.41)					
Post 60	4.13 (0.35)	3.99 (0.45)	4.09 (0.39)	3.78 (0.34)	3.57 (0.31)	3.51 (0.57)	3.25 (0.63)	2.61 (0.65)	3.16 (0.45)					
Ln-SD1, ms										< 0.001	< 0.001	< 0.001	< 0.001	0.050
Pre	3.03 (0.46)	2.88 (0.49)	3.05 (0.43)	2.55 (0.50)	2.59 (0.61)	2.36 (0.59)	1.63 (0.53)	1.72 (0.52)	1.85 (0.50)	0.10	0.14	0.52	(0.08)	0.03
Post 10	3.08 (0.52)	2.17 (0.76)*^,‡,#^	2.66 (0.62)	2.70 (0.57)	1.64 (0.61)*^,‡,#^	2.20 (0.71)	1.68 (0.50)	1.53 (0.74)	1.60 (0.64)					
Post 60	3.21 (0.55)	2.90 (0.71)	3.10 (0.59)	2.70 (0.61)	2.16 (0.75)	2.27 (0.58)	1.75 (0.50)	1.52 (0.59)	1.78 (0.61)					
Ln-SD2, ms										< 0.001	< 0.001	< 0.001	0.003	0.0389
Pre	3.75 (0.39)	3.63 (0.62)	3.93 (0.44)	3.30 (0.32)	3.44 (0.41)	3.23 (0.55)	2.68 (0.57)	2.63 (0.58)	2.82 (0.46)	0.06	0.11	0.53	(0.05)	0.03
Post 10	3.96 (0.40)	3.29 (0.50)*^,‡,#^	3.67 (0.43)	3.41 (0.28)	2.84 (0.70)*^,^^‡^	3.13 (0.58)	2.69 (0.53)	2.40 (0.45)	2.74 (0.44)					
Post 60	3.87 (0.32)	3.73 (0.38)	3.88 (0.37)	3.58 (0.40)	3.14 (0.59)	3.44 (0.41)	3.00 (0.78)	2.37 (0.74)	3.02 (0.49)					
SD1/SD2										< 0.001	< 0.001	< 0.001	0.004	0.040
Pre	0.81 (0.08)	0.80 (0.08)	0.77 (0.07)	0.77 (0.12)	0.75 (0.11)	0.73 (0.12)	0.63 (0.22)	0.67 (0.20)	0.66 (0.20)	0.06	0.06	0.11	0.04	0.03
Post 10	0.78 (0.11)	0.64 (0.16)*^,‡,#^	0.72 (0.11)	0.79 (0.12)	0.56 (0.11)*^,‡^	0.69 (0.14)	0.63 (0.18)	0.65 (0.29)	0.58 (0.20)					
Post 60	0.82 (0.10)	0.77 (0.15)	0.79 (0.09)	0.75 (0.13)	0.67 (0.16)	0.68 (0.10)	0.62 (0.25)	0.65 (0.18)	0.58 (0.15)					

Data presented as mean (SD)

*CON* control, *HIIE* high-intensity interval exercise, *MICE* moderate continuous exercise, *Ex* exercise intervention fixed effect, *Gr* group fixed effect, *IBI* interbeat interval, *SDNN* the standard deviation of NN intervals, *SD1* standard deviation perpendicular to the line of identity of Poincaré plot, *SD2* standard deviation along the line of identity of Poincaré plot, *SD1/SD2* ratio of the derived metrics of the Poincaré plot. Post-hoc comparisons were performed for condition *time interaction separately for each group

*Significantly different from CON (*p* < 0.01)

^‡^Significantly different from pre (*p* < 0.01)

^#^Significantly different from post-60 time-point (*p* < 0.01)

**Table 3:** Acute effects of aerobic exercise on heart rate variability – frequency domain and BEI

	Young adults			Older adults			T2DM			Time	Ex	Group	Ex*Time	Ex*Time*Gr
	CON	HIIE	MICE	CON	HIIE	MICE	CON	HIIE	MICE	*p *(*ω*^2^)	*p *(*ω*^2^)	*p *(*ω*^2^)	*p *(*ω*^2^)	*p *(*ω*^2^)
Ln-LF, ms^2^										< 0.001	< 0.001	0.06	< 0.001	0.045
Pre	6.81 (0.81)	6.60 (1.00)	7.20 (0.70)	5.70 (0.80)	6.05 (1.05)	5.63 (1.08)	4.60 (1.18)	4.30 (1.37)	4.73 (1.01)	0.08	0.14	0. 54	0.07	0.03
Post 10	7.14 (0.87)	5.97 (1.12)*	6.59 0.75)	6.24 (1.13)	4.64 (1.44)^‡^	5.49 (1.46)	4.45 (1.16)	3.49 (1.16)	4.50 (1.09)					
Post 60	7.03 (0.83)	6.84 (0.68)	7.23 (0.65)	6.32 (1.05)	5.39 (1.54)	6.00 (1.14)	4.67 (1.20)	3.80 (1.41)	5.11 (0.91)					
LF, un										0.092	0.023	0.985	0.383	0.193
Pre	54.1 (19.7)	57.2 (14.9)	66.5 (11.1)	53.4 (15.5)	59.0 (15.1)	61.7 (14.6)	67.8 (23.3)	60.0 (25.5)	37.6 (24.2)	0.01	0.02	0.00	0.00	0.00
Post 10	55.8 (14.2)	74.3 (18.2)	64. 3 (18.8)	61.3 (14.7)	67.5 (15.9)	62.8 (18.1)	61.8 (18.8)	54.7 (31.2)	31.3 (16.9)					
Post 60	60.7 (13.3)	64.1 (20.1)	64.8 (14.9)	63.1 (16.1)	63.7 (11.8)	73.0 (15.0)	66.0 (24.2)	57.8 (22.8)	24.6 (15.2)					
HF, un										0.10	.03	0.96	0.36	0.212
Pre	45.9 (19.7)	42.7 (14.9)	33.5 (11.1)	46.6 (16)	36.7 (13.9)	38.3 (14.6)	32.2 (23.3)	40.0 (25.5)	37.6 (24.2)	0.01	0.02)	0.00	0.08	0.00
Post 10	39.4 (13.3)	25.7 (18.2)	35.7 (18.8)	38.7 (18.5)	32.5 (15.9)	37.3 (18.1)	38.2 (18.8)	45.3 (31.2)	31.3 (16.9)					
Post 60	44.2 (14.2)	35.9 (20.1)	35.2 (14.9)	36.9 (16.1)	36.3 (11.8)	27.0 (15.0)	34.0 (24.2)	42.2 (22.8)	24.6 (15.2)					
Ln-LF/HF										< 0.001	< 0.001	< 0.001	< 0.001	0.001
Pre	1.03 (0.14)	1.06 (0.14)	1.11 (0.08)	1.04 (0.14)	1.12 (0.13)	1.12 (0.14)	1.31 (0.41)	1.16 (0.41)	1.18 (0.28)	0.82	0.55	0.24	0.07	0.06
Post 10	1.08 (0.09)	1.36 (0.35)*^,‡^	1.14 (0.18)	1.09 (0.14)	1.29 (0.40)*^,‡^	1.13 (0.19)	1.18 (0.31)	1.17 (0.54)	1.32 (0.31)					
Post 60	1.04 (0.09)	1.13 (0.17)	1.11 (0.11)	1.12 (0.15)	1.13 (0.12)	1.26 (0.18)	1.21 (0.32)	1.14 (0.42)	1.46 (0.47)					
BEI, %										< 0.001	< 0.001	< 0.001	0.004	0.484
Pre	64.0 (8.3)	59.3 (14.8)	55.3 (12.8)	50.4 (15.1)	55.4 (15.9)	47.1 (21.5)	34.0 (21.2)	25.7 (17.2)	34.5 (19.1)	0.04	0.06	0.54	0.04	0.00
Post 10	67.5 (17.0)	46.5 (22.4)*	52.9 (8.2)	52.2 (14.9)	41.8 (25.3)	47.1 (21.5)	31.0 (15.9)	15.8 (13.9)	21.6 (13.2)					
Post 60	63.0 (8.0)	57.3 (19.3)	67.7 (14.9)	54.1 (18.7)	50.1 (18.3)	46.3 (19.2)	26.5 (13.8)	19.7 (15.7)	37.4 (25.7)					

Data presented as mean (SD)

*CON* control, *HIIE* high-intensity interval exercise, *MICE* moderate continuous exercise, *Ex* exercise intervention fixed effect, *Gr* group effect, *LF* low-frequency band, *HF* high-frequency band, *LF/H* the ratio of LF-to-HF power, *BEI* baroreflex effectiveness index. Post-hoc comparisons were performed for condition *time interaction separately for each group

*Significantly different from CON (*p* < 0.01)

^‡^Significantly different from pre (*p* < 0.01)

^#^Significantly different from post-60 measures (*p* < 0.01)

BRS [*F* (2, 33) = 14.85, *p* < 0.001, *ω*^2^ = 0.60] and BEI [*F* (2, 33) = 22.39, *p* < 0.001, *ω*^2^ = 0.54; Table 3] were reduced in participants with T2DM compared to young (BRS, *d* = − 6.81, 95% CI − 9.88 to − 3.74 ms/mmHg, *p* < 0.001; BEI, *d* = − 32.4, 95% CI − 44.56 to 20.32%, *p* < 0.001) and older adults (BRS, *d* = − 3.83, 95% CI − 6.90 to − 0.76, *p* = 0.01; BEI, *d* = − 21.9, 95% CI − 34.0 to − 9.76,* p* < 0.001), but not different amongst participants without T2DM. Group fixed effects for BRS and Ln-RMSSD did not remain significant after adjustment for V̇O_2 peak_, while adjustments for %fat mass only eliminated the BRS group effect. Furthermore, adjustments for resting HR did not change the results of cardiovagal modulation and baroreflex sensitivity. Non-logarithmic cardiovagal modulation data are shown in supplement 2.

### Cardiovagal modulation and baroreflex sensitivity after acute exercise

**Fig. 1:**
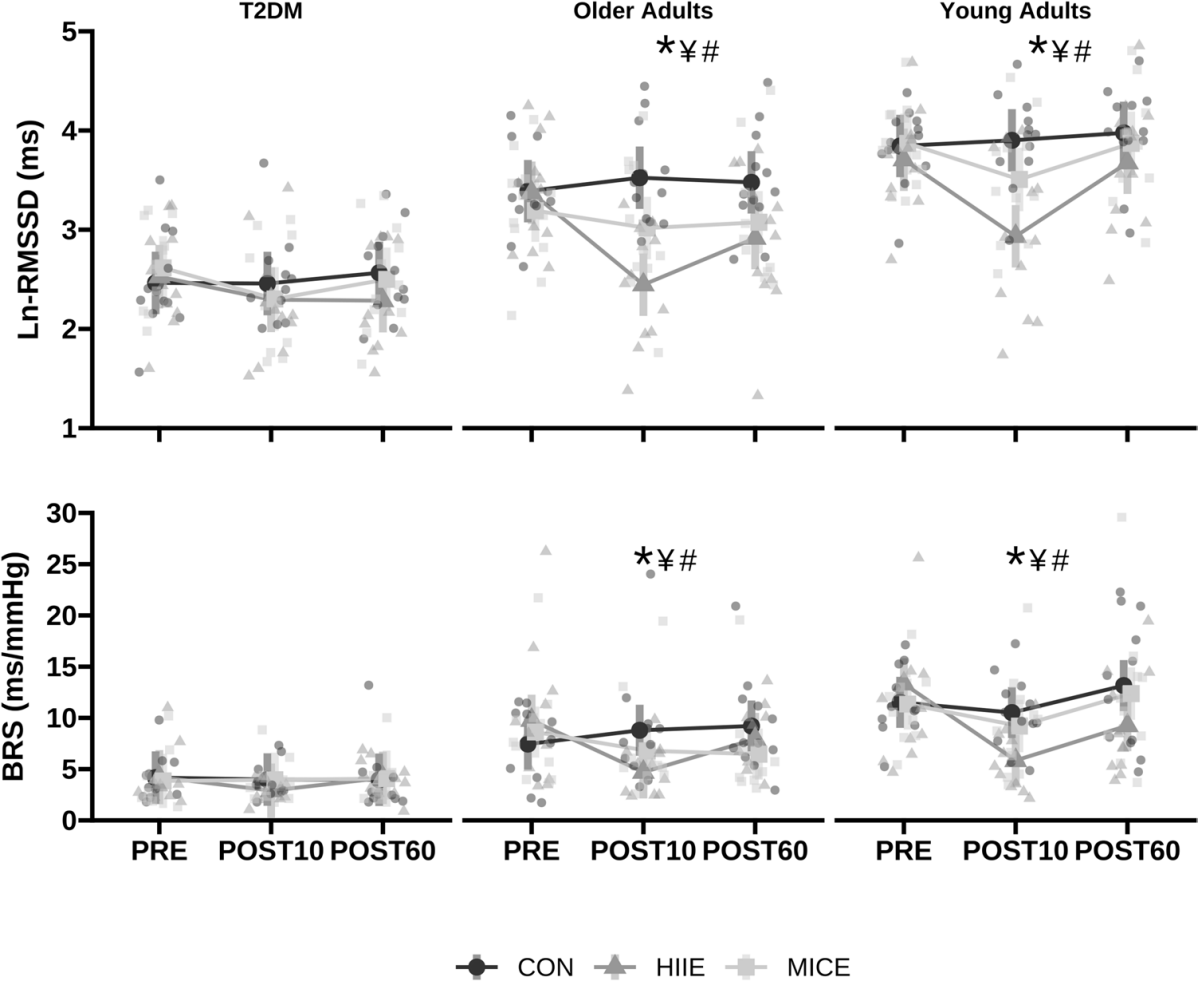
The ln-root mean square of successive differences (RMSSD) and cardiac baroreflex sensitivity post-aerobic exercise response in older adults with and without TD2M and young adults. Vertical bars correspond to the 95% CI. Circles correspond to individual responses in the control (CON) condition. Triangles correspond to individual responses in the high-intensity interval exercise (HIEE) bout, while the squares to the moderate continuous exercise (MICE) bout. *HIIE post 10 significantly different from CON (*p* < 0.01); ^¥^HIIE post 10 significantly different from exercise premeasures (*p* < 0.01); ^#^HIEE post 10 significantly different from exercise post 60 measures (*p* < 0.01)

Exercise-by-time-by-group interaction effects were observed in Ln-SDNN (Table 2), Ln-RMSSD [*F* (8, 254) = 2.065, *p* = 0.039, *ω*^2^ = 0.03], Ln-HF [F (8, 254) = 2.20, *p* = 0.0277, *ω*^2^ = 0.04], Ln-SD1 (Table 2), SD1/ SD2 (Table 2), and BRS [*F* (8, 246) = 3.129, *p* = 0.001, *ω*^2^ = 0.02). This suggests Ln-SDNN (*d* = − 0.53; 95% CI − 0.84 to − 0.23 ms,* p* < 0.001), Ln-RMSSD (*d* = − 0.85: 95% CI − 1.15 to − 0.55 ms, *p* < 0.001) (Fig. 1), Ln-HF (*d* = − 1.60; 95% CI − 2.24 to − 0.97 ms^2^; *p* < 0.001), SD1/SD2 (*d* = − 0.17; 95% CI − 0.25 to − 0.08 ms, *p* < 0.001), and BRS (*d* = − 6.32; 95% CI − 9.35 to − 3.29 ms/mmHg, *p* < 0.001) (Fig. 1) decreased immediately following HIIE in both young and older adults but not in participants with T2DM. Only the interbeat interval (IBI) was reduced after HIIE in both participants with and without T2DM (Table 2). These outcomes returned to baseline following a 60-min recovery period. Adjustments for V̇O_2__peak_, HRpeak and %fat mass did not change the results, while adjustment for IBI abolished the significant interactions described above. Cardiovagal modulation and BRS indices remained unchanged after MICE. Normalized frequency HRV outcomes are depicted in Table 3.

## Discussion

The main findings of the present study were that cardiovagal modulation remained unchanged in older adults with T2DM following a single bout of HIIE and MICE, whereas in young and older adults without T2DM cardiovagal modulation was reduced 10-min after HIIE. All indices of cardiovagal modulation returned to baseline levels 60 min into recovery. These findings support our initial hypothesis that cardiovagal modulation following an acute aerobic exercise is disease- and intensity-dependent, but apparently not influenced by age.

### Cardiovagal modulation at rest

In this study, cardiovagal modulation indices in older adults with T2DM were markedly reduced compared to young (Ln-RMSSD: − 34%, Ln-HF: − 40%) and older adults without T2DM (Ln-RMSSD: − 22%, Ln-HF: − 27%). This is in line with observations from cross-sectional and prospective cohort studies suggesting that the incidence of cardiac autonomic dysfunction increases with age and may contribute to the pathogenesis of T2DM, but also that T2DM may cause imbalances in cardiovagal modulation (Gerritsen et al. 2001; Carnethon et al. 2003; Almeida-Santos et al. 2016). In fact, only one adult with T2DM in our sample would not meet the criteria for CAN based on recent cut-off values for resting HRV measurements (e.g., RMSSD < 26.18 ms) (Bhati et al. 2019). Even if the authors concluded that cardiovagal modulation measurements at rest were less sensitive to detect CAN compared to post-maximal exercise measurements, resting values in older adults with T2DM in the present study were similar to the proposed cut-off values for post-exercise HRV measurements (i.e., RMSSD: ~ 14 ms vs < 14.85 ms) (Bhati et al. 2019). This suggests that the incidence of CAN in older adults with TDM2 is higher, likely due to prolonged hyperglycemia causing damage to the vagus nerve and hence contributing to cardiac autonomic dysfunction (Vinik et al. 2013), even though they have adequate glycemic control. Mechanistically, longstanding hyperglycemia increases protein glycation causing accumulation of advanced glycation end products (AGEs) and upregulates reactive oxygen species production through its receptor’s signaling, which ultimately activates proinflammatory responses mediated by nuclear kB factor leading to cytotoxic damage (Vinik et al. 2013). In addition, the burden of CAN is expected to arise secondarily to increases in sympathetic outflow to the heart and vasculature from the insulin therapy prescribed as β-cells become progressively dysfunctional with advanced T2DM (Paolisso et al. 1999; Muta et al. 2015). Age-related catecholamine spill-over may also increase insulin resistance in pre-diabetic older adults and worsen CAN in T2DM, respectively, as catecholamines promote free-fatty acids and glucose release into circulation (Benthem et al. 2000).

### Cardiovagal modulation responses after aerobic exercise

The intensity of aerobic exercise is the main determinant of early cardiovagal modulation recovery in healthy physically active young and older adults (Michael et al. 2017a), but not in middle-aged and older adults with T2DM (Figueroa et al. 2006, 2007). Reductions in indices of cardiovagal modulation and BRS have been reported following a bout of submaximal or maximal aerobic exercise in middle-aged and older adults with T2DM (Figueroa et al. 2006; Banthia et al. 2013; Bhati et al. 2019; Goldberger et al. 2022), while in young and older adults, these appear mainly after near-maximal exercise (Michael et al. 2017a). However, in the present study, the indices of cardiovagal modulation and BRS remained unchanged in older adults with TDM2 after HIIE and MICE. Sex and methodological differences between studies in exercise intensity, duration of the exercise, or the time points at which HRV was measured during recovery, are likely candidates to explain this discrepant finding. For instance, in the study by Figueroa et al. (Figueroa et al. 2006) enrolling 8 middle-aged women with obesity and T2DM, 20-min of walking (~ 65% of V̇O_2__peak_) was sufficient to reduce post-exercise HF and BRS, whereas in this study, older male adults with T2DM exercised 40% longer and still no changes in HF and BRS were observed. We assessed post-exercise cardiovagal modulation and BRS 10 min into recovery which could have resulted in an inability to detect changes appearing outside this time-point (< 10 min). However, this is also a doubtful explanation as reductions in cardiovagal modulation have been reported to occur between 3 and 10 min after exercise cessation and remain reduced 20 to 30 min into recovery in middle-aged and older adults with T2DM (Figueroa et al. 2006; Banthia et al. 2013; Bhati et al. 2019; Goldberger et al. 2022). In addition, a slower cardiovagal recovery is generally observed in adults with low CRF compared to fit adults, as was the case with our adults with and without T2DM, respectively (Stanley et al. 2013). Another explanation may be that the loss of cardiovagal predominance at rest in older adults with T2DM may transpose into the early recovery period, in line with the findings suggesting that both resting and post-exercise HRV provide an accurate diagnostic test for CAD in T2DM (Sacre et al. 2012). Finally, changes in indices of cardiovagal modulation following exercise should be interpreted with caution, as they may simply reflect a mathematical artefact, given the non-linear association between HR (or IBI) and HRV (Boyett et al. 2013, 2017; Sacha et al. 2013). In fact, as we covaried indices of HRV and BRS to HR as suggested by Sacha et al. (2013), changes in indices of HRV and BRS observed 10-min after HIIE in young and older adults were no longer significant, although group differences were still observed. This may suggest that changes in cardiovagal modulation after exercise are mediated by HR recovery to a greater extent than HR influences cardiovagal modulation at rest.

### Limitations

The present study is not without limitations. Respiratory sinus arrhythmia was not controlled and may have influenced cardiovagal modulation and BRS estimates. Although paced breathing has been suggested to reduce respiratory influences on autonomic parameters (Shaffer and Ginsberg 2017), research shows that it does not significantly affect post-exercise cardiovagal modulation (Kaikkonen et al. 2007). BRS was assessed with the spontaneous sequence method which may not reflect the “true” sensitivity of the arterial baroreflex arch to pharmacological and mechanical stressors as measured with the Oxford and the neck chamber methods, respectively, in fact poor agreement has been reported between methods (Lipman et al. 2003). Thus, findings from studies using different baroreflex assessment methodologies should be interpreted with caution. The lack of invasive measures in this study, such as catecholamine plasma concentrations and blood lactate, precluded meaningful mechanistic insight into sympathetic activity and skeletal muscle recovery after exercise. Furthermore, the indices of cardiovagal modulation and BRS dynamics during exercise were not measured, excluding additional inferences. Most participants with T2DM were taking oral glycemic agents, and insulin therapy, which may have confounded the present findings. It would have been ideal to take these participants off their medication, but ethical and medical considerations must be considered, particularly in the presence of prolonged T2DM. We have not assessed CAN using the gold standard cardiovascular autonomic reflex tests (CARTs)—Ewing’s criteria—and as such, we were unable to truly confirm CAN within participants with T2DM (Ewing et al. 1985). Finally, our results apply only to older male adults with and without T2DM and male young adults without T2DM. Young and older adult females with and without TD2M can show distinct post-exercise cardiac autonomic recovery patterns, thus limiting the generalization of our results.

## Conclusions

The main findings of the present study were that cardiovagal modulation remained unchanged in older adults with T2DM following a single bout of HIIE and MICE, whereas in young and older adults without T2DM cardiovagal modulation was reduced 10-min after HIIE. All indices of cardiovagal modulation returned to baseline levels 60 min into recovery. Overall, these findings support our initial hypothesis as cardiovagal modulation following an acute aerobic exercise is disease- and intensity-dependent, but apparently not influenced by age.

### Supplementary Information

Below is the link to the electronic supplementary material.

Supplementary file1 (DOCX 40 KB)

Supplementary file2 (TIF 133 KB)

## Data Availability

The data that support the findings of this study are available from the corresponding author, XM, upon reasonable request.
