# Supplementary material for: Post-acute exercise cardiovagal modulation in older male adults with and without type 2 diabetes
Source: Eur J Appl Physiol. 2023 Dec 20;124(5):1475–86. doi: 10.1007/s00421-023-05357-3 (PMC11055715; doi:10.1007/s00421-023-05357-3)
Supplement: Supplementary file 1 — Supplementary file1 (DOCX 40 KB) [file 421_2023_5357_MOESM1_ESM.docx]

|  | **Young Adults** | | | **Older Adults** | | | | **T2DM** | | | *Time* | *Ex* | *Group* | *Time*Ex* |
| --- | --- | --- | --- | --- | --- | --- | --- | --- | --- | --- | --- | --- | --- | --- |
|  | CON | HIIE | MICE | CON | HIIE | MICE | CON  CON  CON | | HIIE | MICE | *p (ω^2^)*  CON | *p (ω^2^)* | *p (ω^2^)* | *p (ω^2^)* |
| *IBI, ms* |  |  |  |  |  |  |  | | | |  | | | |
| Pre | 1020 (156) | 1045 (131) | 1020 (115) | 1093 (148) | 1061 (136) | 1021 (151) | 906 (120) | | 901 (122) | 915 (156) | **< .001** (.31) | **<.001** (.34) | .06 (.12) | **<.001** (.21) |
| Post 10 | 990 (127) | 728 (234)* ⱡ# | 890 (133)* ⱡ | 1103 (125) | 812 (111)* ⱡ | 910 (129) *ⱡ | 920 (128) | | 762 (117)* ⱡ | 763 (245)* ⱡ |  |  |  |  |
| Post 60 | 1023 (142) | 893 (112) | 997 (115) | 1072 (116) | 879 (143) | 943 (130) | 925 (131) | | 808 (129) | 863 (130) |  |  |  |  |
| *SDNN, ms* |  |  |  |  |  |  |  | | | |  | | | |
| Pre | 55.14 (16.22) | 57.32 (36.63) | 73.43 (37.46) | 37.00 (11.47) | 43.15 (15.77) | 36.93 (17.06) | 21.47 (10.28) | | 19.73 (8.88) | 21.85 (8.27) | **.002** (.04) | **<.001** (.06) | **<.001** (0.51) | **.003** (.06) |
| Post 10 | 73.80 (36.42) | 35.63 (15.16)* ⱡ# | 56.07 (35.68) | 42.96 (13.32) | 25.05 (14.50))* ⱡ | 31.94 (12.29) | 20.30 (10.56) | | 15.25 (6.35) | 20.67 (7.54) |  |  |  |  |
| Post 60 | 65.53 (20.68) | 59.34 (27.71) | 64.35 (25.50) | 46.50 (17.14) | 37.76 (22.30) | 38.60 (20.63) | 30.57 (19.15) | | 16.50 (10.69) | 25.66 (10.99) |  |  |  |  |
| *RMSSD,ms* |  |  |  |  |  |  |  | | | |  | | | |
| Pre | 49.69 (16.37) | 45.59 (24.11) | 52.05 (23.03) | 32.47 (15.05) | 33.71 (19.24) | 27.76 (14.71) | 13.32 (7.72) | | 13.92 (9.94) | 15.26 (6.30) | **.001** (.06) | **<.001** (.12) | **<.001** (.48) | **<.001** (.06) |
| Post 10 | 54.05 (23.17) | 24.38 (17.49)* ⱡ# | 39.56 (24.81) | 38.49 (21.91) | 13.35 (7.07)* ⱡ | 24.82 (16.52) | 13.62 (9.02) | | 11.53 (8.56) | 11.79 (6.45) |  |  |  |  |
| Post 60 | 58.61 (24.53) | 47.74 (31.57) | 55.20 (31.44) | 36.95 (21.33) | 22.49 (13.28) | 26.64 (21.67) | 14.32 (6.77) | | 11.03 (7.04) | 14.31 (7.76) |  |  |  |  |
| *SD1, ms* |  |  |  |  |  |  |  | | | |  | | | |
| Pre | 22.46 (8.60) | 20.02 (11.63) | 23.2 (11.01) | 14.53 (8.70) | 15.79 (10.39) | 12.26 (6.77) | 5.94 (4.17) | | 6.30 (3.40) | 7.10 (3.60) | **.001** (.04) | **.001** (.07) | **.001** (.44) | **.02** (.03) |
| Post 10 | 24.32 (12.10) | 11.27 (8.13)* ⱡ# | 17.01 (11.04) | 17.60 (11.64) | 6.05 (3.63)* ⱡ | 11.29 (7.90) | 6.14 (4.24) | | 6.19 (6.01) | 6.02 (4.14) |  |  |  |  |
| Post 60 | 27.75 (12.77) | 23.26 (19.70) | 25.70 (14.52) | 18.06 (13.59) | 10.86 (6.90) | 11.57 (8.67) | 6.45 (3.21) | | 5.36 (3.24) | 7.12 (4.78) |  |  |  |  |
| *SD2, ms* |  |  |  |  |  |  |  | | | |  | | | |
| Pre | 45.23 (15.77) | 44.99 (28.73) | 55.70 (25.81) | 28.30 (9.56) | 33.58 (12.92) | 28.79 (14.63) | 16.71 (8.72) | | 15.79 (7.53) | 18.29 (7.71) | **.002** (.04) | **<.001** (.07) | **<.001** (.52) | **.003** (.05) |
| Post 10 | 56.94 (27.24) | 29.74 (13.11)* ⱡ | 43.41 (24.87) | 31.49 (9.64) | 20.92 (13.81)* ⱡ | 25.72 (9.95) | 16.71 (8.88) | | 11.96 (4.79) | 16.81 (6.92) |  |  |  |  |
| Post 60 | 49.99 (15.26) | 44.35 (17.45) | 51.28 (18.33) | 38.88 (17.54) | 26.39 (12.67) | 30.85 (15.79) | 25.81 (19.52) | | 13.41 (8.75) | 22.83 (10.58) |  |  |  |  |
| *SD1/SD2* |  |  |  |  |  |  |  | | | |  | | | |
| Pre | 0.50 (0.14) | 0.50 (0.18) | 0.43 (0.12) | 0.52 (0.26) | 0.45 (0.18) | 0.45 (0.19) | 0.44 (0.34) | | 0.46 (0.28) | 0.45 (0.37) | **<.001** (.05) | **<.001** (.07) | **<.001** (.46) | **.02** (.03) |
| Post 10 | 0.45 (0.18) | 0.36 (0.18) | 0.38 (0.12) | 0.53 (0.20) | 0.31 (0.10) | 0.43 (0.19) | 0.41 (0.25) | | 0.55 (0.47) | 0.37 (0.22) |  |  |  |  |
| Post 60 | 0.54 (0.18 | 0.52 (0.38) | 0.47 (0.14) | 0.45 (0.19) | 0.40 (0.12) | 0.38 (0.14) | 0.40 (0.41) | | 0.47 (0.23) | 0.32 (0.14) |  |  |  |  |
| Data presented as mean (SD); Abbreviations: CON, control; HIIE, high-intensity interval exercise; MICE, moderate continuous exercise, IBI, interbeat interval; SDNN, standard deviation of NN intervals; SD1 standard deviation perpendicular to the line of identity of Poincaré plot; SD2, standard deviation along the line of identity of Poincaré plot. Post-hoc comparisons were performed for condition *time interaction separately for each group * Significantly different from CON (*p* < 0.05). ⱡ Significantly different from pre (*p* < 0.05). # Significantly different from post 60 measures (*p* < 0.05) | | | | | | | | | | | | | | |

|  | **Young Adults** | | | **Older Adults** | | | | **T2DM** | | | *Time* | *Ex* | *Group* | *Time*Ex* |
| --- | --- | --- | --- | --- | --- | --- | --- | --- | --- | --- | --- | --- | --- | --- |
|  | CON | HIIE | MICE | CON | HIIE | MICE | CON  CON  CON | | HIIE | MICE | *p (η^2^)*  CON | *p (η^2^)* | *p (η^2^)* | *p (η^2^)* |
| *LF, ms^2^* |  |  |  |  |  |  |  | | | |  | | | |
| Pre | 1164 (776) | 1213 (1567) | 1672 (1277) | 397 (321) | 641 (486) | 421 (345) | 172 (205) | | 129 (114) | 171 (152) | .06 (.31) | **.001** (.05) | **<.001** (.45) | **.001** (.07) |
| Post 10 | 1745 (1454) | 617 (490) * ⱡ | 895 (520) | 945 (1188) | 202 (222) | 501 (588) | 155 (183) | | 53 (53) | 135 (99) |  |  |  |  |
| Post 60 | 1451 (933) | 1161 (839) | 1643 (979) | 968 (1141) | 486 (550) | 686 (694) | 168 (132) | | 97 (119) | 240 (218) |  |  |  |  |
| *HF, ms^2^* |  |  |  |  |  |  |  | | | |  | | | |
| Pre | 947 (549) | 763 (682) | 808 (532) | 348 (305) | 406 (436) | 268 (297) | 70 (95) | | 73 (71) | 93 (97) | **.03** (.03) | **.006** (.04) | **<.001** (0.43) | .172 (.08) |
| Post 10 | 1040 (804) | 303 (377) | 666 (771) | 487 (589) | 87 (88) | 295 (423) | 80 (118) | | 55 (72) | 66 (67) |  |  |  |  |
| Post 60 | 1108 (717) | 870 (1026) | 1277 (1744) | 514 (658) | 246 (263) | 384 (760) | 68 (57) | | 46 (40) | 85 (97) |  |  |  |  |
| *LF/HF,ms^2^* |  |  |  |  |  |  |  | | | |  | | | |
| Pre | 1.59 (1.14) | 1.66 (1.11) | 2.25 (0.94) | 1.45 (1.09) | 2.23 (1.50) | 2.19 (1.74) | 4.15 (4.00) | | 2.65 (2.30) | 2.79 (2.07) | **.04** (.02) | **.04** (.03) | .367 (.06) | **.003** (.06) |
| Post 10 | 1.91 (1.27) | 4.74 (3.41) *ⱡ | 2.90 (2.83) | 2.16 (1.42) | 2.49 (1.96) | 2.49 (1.96) | 2.98 (3.65) | | 2.58 (2.56) | 3.54 (3.10) |  |  |  |  |
| Post 60 | 1.53 (0.93) | 2.56 (1.71) | 2.22 (1.06) | 2.52 (2.21) | 2.01 (0.86) | 4.23 (3.51) | 3.21 (2.38) | | 2.25 (2.04) | 5.644 (5.7) |  |  |  |  |
| Data presented as mean (SD); Abbreviations: CON, control; HIIE, high-intensity interval exercise; MICE, moderate continuous exercise, IBI, interbeat interval; SDNN, standard deviation of NN intervals; SD1 standard deviation perpendicular to the line of identity of Poincaré plot; SD2, standard deviation along the line of identity of Poincaré plot. Post-hoc comparisons were performed for condition *time interaction separately for each group * Significantly different from CON (*p* < .05). ⱡ Significantly different from pre (*p* < .05). # Significantly different from post 60 measures (*p* < .05) | | | | | | | | | | | | | | |
