# Supplementary figures and images for: Post-acute exercise cardiovagal modulation in older male adults with and without type 2 diabetes
Source: Eur J Appl Physiol. 2023 Dec 20;124(5):1475–86. doi: 10.1007/s00421-023-05357-3 (PMC11055715; doi:10.1007/s00421-023-05357-3)

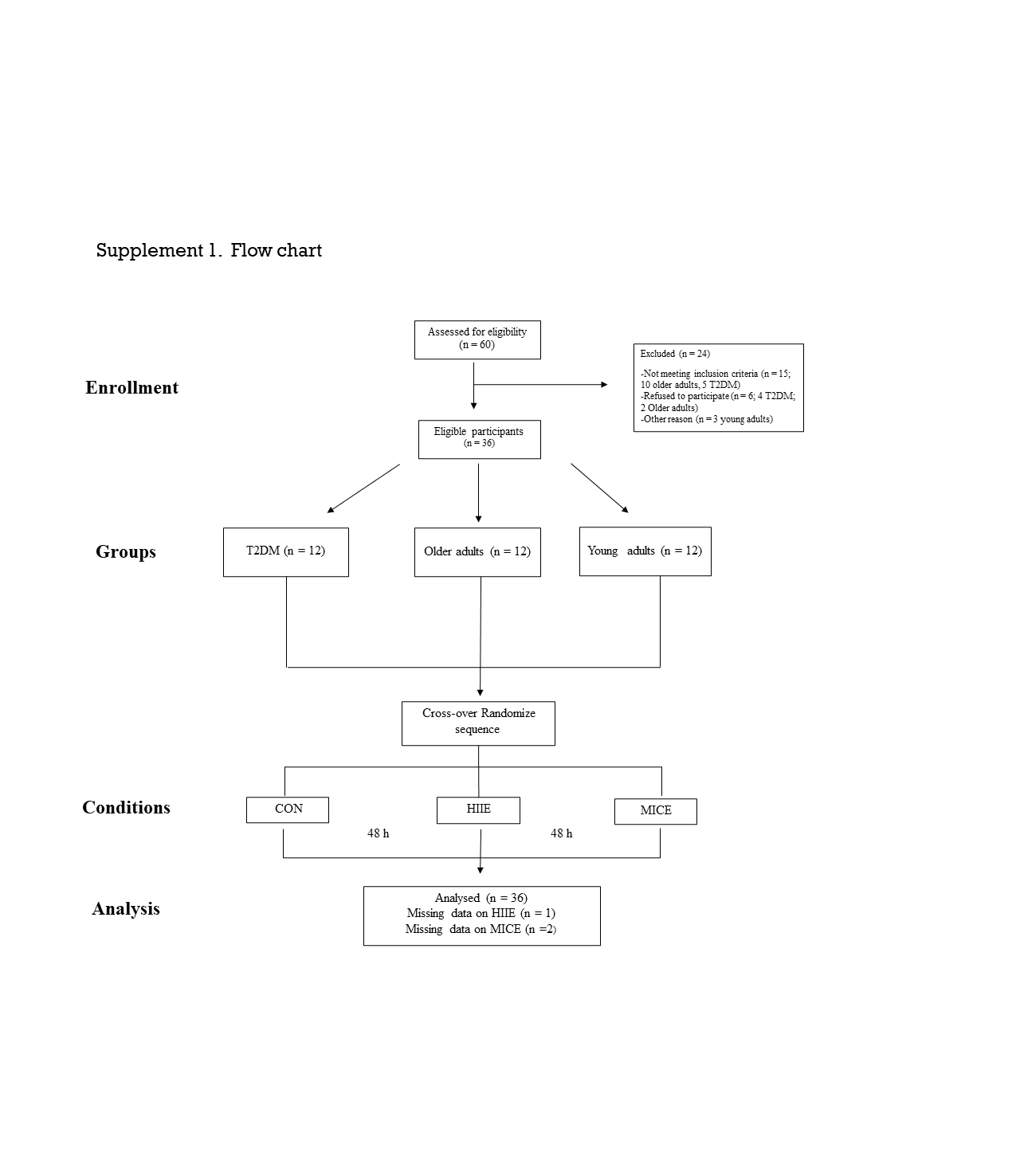

Supplement: Supplementary file 2 — Supplementary file2 (TIF 133 KB) [file 421_2023_5357_MOESM2_ESM.tif]
